# Molecular Basis of Synergistic Causal Effect of Dual GLP-1R and GIPR Agonists for Risk Reduction in Diabetic Retinopathy, Alzheimer Disease, and Coronary Artery Disease in Diabetic Patients

**DOI:** 10.3390/genes17060602

**Published:** 2026-05-25

**Authors:** Jiehui Xu, Yibeltal A. Ashebir, Yongzhao Shao

**Affiliations:** Department of Population Health, NYU Grossman School of Medicine, New York, NY 10016, USA; jx2184@nyu.edu (J.X.); yibeltal.ashebir@nyulangone.org (Y.A.A.)

**Keywords:** Alzheimer’s diseases, diabetic retinopathy, coronary artery disease, antidiabetic drug, synergistic causal effect, cis-MR, multivariate Mendelian Randomization, PC-GMM

## Abstract

**Background:** The dual agonism of glucagon-like peptide-1 receptor (GLP-1R) and glucose-dependent insulinotropic polypeptide receptor (GIPR) becomes a groundbreaking treatment for type 2 diabetes (T2D) that achieves robust glycemic control and maintains body weight. It also induces potential risk reduction in diabetic retinopathy (DR), Alzheimer disease (AD), and heart diseases including coronary artery disease (CAD) in treated T2D patients. To date, the molecular basis underpinning the remarkable causal treatment effects and synergy of the dual agonism of GLP-1R and GIPR on risk reduction in T2D, CAD, DR and AD has not been systematically investigated. **Methods:** To elucidate the treatment effects and potential synergy of dual GLP-1R/GIPR agonism on risk reduction in T2D, CAD, DR and AD while minimizing the impact of confounders, we used a robust cis-Mendelian randomization (cis-MR) with a principal component-based generalized method of moments (PC-GMM) where blood-based glycated hemoglobin (HbA1c), high- and low-density lipoprotein cholesterol (HDL-c, LDL-c), and BMI were used as mediating biomarkers. **Results:** Our cis-MR analyses confirmed a synergistic causal protective effect of dual GLP-1R/GIPR agonism on T2D via HbA1c reduction [OR = 0.17; 95% CI = (0.11, 0.26); *p* = 3.68 × 10^−17^] which is more significant than either GLP-1R agonism or GIPR agonism alone. Similarly, the causal protective effect of dual GLP-1R/GIPR agonism via HbA1c reduction was also significant for DR [OR = 0.20; 95% CI = (0.11, 0.36); *p* = 9.22 × 10^−8^]. Further, our multivariate cis-MR (or cis-MVMR) analyses revealed that after adjusting for HbA1c, a synergistic protective effect on DR via a reduction in LDL-c is significant in dual GLP-1R/GIPR agonism [OR = 0.57; 95% CI = (0.29, 0.94)], while the protective effect on DR of LDL-c reduction is non-significant in either GLP-1R agonism or GIPR agonism alone. Also, after adjusting for HbA1c, the multivariate cis-MR results showed significant protective effects on AD via a reduction in LDL-c in GLP-1R/GIPR agonism [OR = 0.44; 95% CI = (0.25, 0.81)]. Importantly, the multivariate cis-MR results also revealed that dual GLP-1R/GIPR agonism has significant protective effects on CAD via both a reduction in BMI [OR = 0.46; 95% CI = (0.28, 0.75)] and an improvement in HDL [OR = 0.59; 95% CI = (0.39, 0.90)]. This is in support of the hypothesis that dual GLP-1R/GIPR agonism has a synergistic protective effect on CAD that is stronger than that of GLP-1R agonism alone, which yielded a non-significant causal effect for both HDL and BMI, and GIPR agonism alone also yielded a non-significant causal effect for HDL when adjusted for BMI. **Conclusions:** These novel findings have significant implications for repurposing dual incretin agonism in terms of diabetic drugs to serve as a unifying, precision prevention strategy against CAD, DR and AD as leading drivers of mortality and morbidity in diabetic patients.

## 1. Introduction

The recent global escalation in obesity has reached epidemic proportions, serving as the principal predisposing risk factor for type 2 diabetes (T2D), which is now a worldwide health concern [1]. Patients with T2D face an elevated risk of developing diabetic retinopathy (DR), a retinal pathology leading to vision loss, which is the most prevalent microvascular complication and is fundamentally linked to neurovascular decay [2,3]. Crucially, diabetes acts as a major systemic driver for DR and related neurodegeneration. Alzheimer’s disease (AD), as the leading cause of dementia and related mortality globally, is profoundly exacerbated by diabetic pathology, to the extent that it is frequently referred to as “type 3 diabetes” [4,5]. Large-scale national cohort studies report that DR, in particular, is a leading risk factor and clinical precursor for AD in diabetic patients, suggesting a shared pathological axis of chronic inflammation, oxidative stress, and microvascular decline [2,6]. Currently, AD lacks a cure or effective disease-modifying therapeutic options and broadly effective preventive measures due to its extensive etiologic heterogeneity [7,8,9,10,11]. Consequently, there is an urgent need to identify precision prevention strategies for AD, particularly within vulnerable populations suffering from T2D and its vascular complications.

Parallel to these microvascular threats, coronary artery disease (CAD) is one of the life-threatening macrovascular complications seen in T2D patients [1] and a leading cause of global mortality [12]. Accelerated by atherogenic dyslipidemia, specifically imbalances between low-density lipoprotein cholesterol (LDL-c) and high-density lipoprotein cholesterol (HDL-c), as well as elevated body mass index (BMI), CAD is a major driver of premature mortality in patients with diabetes [13,14]. Consequently, targeted metabolic and lipidomic interventions are critical for extending survival in this population.

Given their primary function of enhancing insulin signaling and regulating glucose metabolism, antidiabetic drugs have long been viewed as potential candidates for drug repurposing to prevent diabetes-related complications, including cardiovascular mortality and dementia. Recently, the dual agonism of the glucagon-like peptide-1 receptor (GLP-1R) and the glucose-dependent insulinotropic polypeptide receptor (GIPR) has emerged as a cornerstone in the treatment of T2D and obesity [15]. Agents in this class demonstrate profound efficacy in lowering glycated hemoglobin (HbA1c) and body mass index (BMI) [16,17] while simultaneously improving broader lipidomic profiles, including a reduction in low-density lipoprotein cholesterol (LDL-c) and an elevation in high-density lipoprotein cholesterol (HDL-c) [18,19].

Beyond systemic metabolic homeostasis, these incretin receptors are widely expressed in the central nervous system, particularly in regions critical for memory and cognition, such as the hippocampus and frontal cortex [20,21]. Extensive preclinical evidence indicates that incretins readily cross the blood–brain barrier to regulate synaptic plasticity, mitigate neuroinflammation, and restore neuronal bioenergetics [21,22]. Building on this neurometabolic link, recent real-world evidence has substantiated the neuroprotective potential of incretin therapies, demonstrating that GLP-1R agonists are associated with a significantly reduced risk of AD compared to other antidiabetic agents [23,24,25].

Despite the proven clinical efficacy of GLP-1R and GIPR dual agonism on T2D and its observed observational associations with AD and DR risk reduction, the causal molecular mechanisms driving these pleiotropic benefits remain largely uncharacterized. First, existing studies evaluating AD risk primarily focus on the protective effects of glycemic (HbA1c) control, often ignoring the causal contributions of lipidomic modulation (e.g., LDL-c). The 2024 Lancet Commission recently identified elevated LDL-c as a leading modifiable risk factor for dementia [26]. Thus, an important open question is whether dual GLP-1R and GIPR agonism can exert synergistic causal protection against DR and AD via LDL-c reduction. Second, existing studies have not assessed the statistical significance of these pathways; it is unknown whether the protective effect of lowering LDL-c on DR and AD remains significant when rigorously adjusted for concurrent HbA1c reductions. Third, existing human population studies and trials produced inconsistent results and do not provide mechanistic insights into the causal effect of dual agonism on risk reduction for AD. There are extensive and in-depth discussions deliberating the synergistic effect of dual GLP1-R/GIPR agonists on T2D and obesity but a lack of discussion on the synergistic effect on risk reduction for DR, AD, and/or CAD [15]. Forth, experimental animal mechanistic studies do not fully reflect the extensive heterogeneity of aging human populations with diverse age-related comorbidities and may not be able to capture the causal effect under long-term or lifetime exposure. To fill in these knowledge gaps, Mendelian randomization (MR) studies that utilize genetic variants at birth as instrumental variables can potentially elucidate causal relationships while minimizing biases from confounding and reverse causation. However, existing two-sample MR studies were not focused on assessing the causal effects of dual GLP-1R/GIPR agonism and did not explore potential synergistic effects on DR, AD and CAD [27,28,29,30]. Other MR studies generally used the genome-wide selection of genetic variants as instrumental variables and are therefore prone to severe biases due to unadjusted pleiotropic effects [31,32].

To address these critical knowledge gaps, we employed a robust cis-Mendelian randomization (cis-MR), utilizing genetic variants strictly localized to GLP1R and GIPR drug target coding regions to mimic targeted pharmacological perturbation. This approach inherently minimizes the widespread horizontal pleiotropy that commonly confounds traditional MR studies. Specifically, we leveraged multivariable cis-MR (cis-MVMR) to simultaneously disentangle the distinct causal effects of HbA1c and LDL-c on DR and AD and those of BMI and HDL-c on CAD. These analyses were implemented using a robust principal component-based generalized method of moments estimator (PC-GMM), which derives robust effect estimates from highly correlated instruments within the drug target coding gene region (cis-MR PC-GMM) [33,34]. For example, GLP1R agonists have proven efficacy for T2D and obesity [15] and for preventing cardiovascular events in T2D patients [35,36,37,38,39,40]. Using robust PC-GMM-based cis-MVMR, Patel et al. (2024) showed that GLP1R agonism has a significant protective effect for CAD by controlling BMI when its effect on T2D was adjusted [33]. However, there has been no study on the molecular basis underlying the potential synergy and causal effect of dual GLP1R/GIPR agonism on CAD. Given that cardiovascular risk is a major mortality driver in T2D patients, it is of great interest to investigate whether dual GLP1R/GIPR agonism’s maintenance of HDL-c homeostasis has a significant synergistic effect on reducing the risk of CAD when adjusted for BMI in the sense that dual agonism has a significant protect effect that is more significant than that of individual agonism alone. By using *GLP1-R*- and *GIPR*-localized genetic proxies to mimic pharmacological perturbation in a robust cis-MVMR, this study aims to systematically elucidate whether the shared and distinct molecular pathways through which dual incretin targeting confers synergistic, protective effects against the intersecting clinical burden of T2D, DR, AD and CAD.

## 2. Materials and Methods

### 2.1. Study Design

We employed a robust cis-Mendelian randomization (MR) framework to investigate the causal effects of dual-targeting antidiabetic drug-induced alteration in lipid/glucose/ anthropometric profiles on disease risk for Alzheimer’s disease (AD), diabetic retinopathy (DR) and coronary artery disease (CAD), as proxied by genetic variations near the coding regions of the drug targets GLP1-R and GIPR.

**Figure 1:**
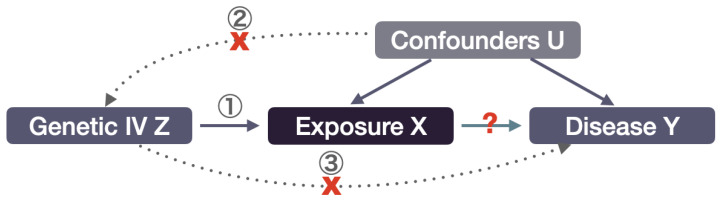
**A directed acyclic graph (DAG) for core IV assumptions in Mendelian randomization.** The solid arrows indicate naturally existing or assumption-required associations, and dotted lines marked with a red ”X” indicate associations that must not exist, with numbers in correspondence to IV assumptions: (1) the relevance assumption, (2) the independence assumption, and (3) the exclusion restriction assumption. The question mark denotes the possible causal association from exposure to the disease outcome, which is the relationship that MR analysis aims to estimate.

The validity of the MR analysis rests upon three core instrumental variable (IV) assumptions, as illustrated in Figure 1: (1) the relevance assumption, where IVs are strongly associated with exposure; (2) the independence assumption, where IVs are not associated with confounders; and (3) the exclusion restriction assumption, where IVs influence the outcome exclusively through exposure. In our study, we adopted a cis-MR design and limited IVs within the drug target genes *GLP1-R*/*GIPR* to better proxy dual-targeting drug influence via the agonism of the target gene products, thereby minimizing potential bias from horizontal pleiotropy and reducing the inherent multi-gene heterogeneity in MR designs.

The overall study design is aimed at investigating the molecular basis underlying joint causal effects and substantiating the potential synergy of the dual agonism of GLP1-R/GIPR for enhanced risk reduction in DR, AD and CAD among T2D patients.

First, to validate the instrumental variables (IVs) and the robust PC-GMM framework, we performed positive control analyses leveraging the established clinical relationship between glycated hemoglobin (HbA1c) and type 2 diabetes (T2D). Given that elevated HbA1c is a clinical diagnostic standard for T2D, a synergistic significant causal association for both GLP-1R and GIPR agonism is expected due to the clinical synonymity between chronic hyperglycemia and the diagnosis of T2D. In particular, we employed a two-sample univariable cis-Mendelian randomization (cis-UVMR) analysis to examine this expected causality, using genetic variants within the *GLP1-R* and *GIPR* regions as drug target instrumental variables (IVs) and top principal components accounting for 99% of the weighted genetic variation and generalized method of moments (GMM) for the estimation of the effects.

Second, following the positive control in assessing the potential synergistic effect of dual GLP1-R/GIPR agonism on T2D, we subsequently implemented a univariable cis-Mendelian randomization (cis-UVMR) approach using the robust PC-GMM estimator of Patel et al. [33] to identify potential causal pathways linking the *GLP1-R* and *GIPR* loci to Alzheimer’s disease (AD), diabetic retinopathy (DR) and coronary artery disease (CAD), with potential synergistic effects. For DR and AD, we specifically focused on HbA1c and LDL-c as mediating biomarkers, as these represent the primary glycemic and lipidomic pathways through which incretin mimetics are clinically expected to exert neurovascular protection. For CAD, we examined BMI given its established causal association via the *GLP1R* locus [33] and HDL-c due to its well-known role in mitigating atherosclerotic risk. Evaluating these specific exposures allows us to comprehensively assess the systemic macrovascular impact of these loci.

Third, to further disentangle the related causal contributions of glycemic and lipidomic pathways, we conducted a multivariable cis-Mendelian randomization (cis-MVMR) analysis using PC-GMM. While the preceding cis-UVMR provided a view of individual biomarker effects, the cis-MVMR framework provides crucial further insights in this context by accounting for potential horizontal pleiotropy and the inherent genetic correlation between HbA1c and LDL-c within the *GLP1R* and *GIPR* loci. By evaluating these exposures simultaneously, cis-MVMR identifies the disentangled, “direct” effect of each mediator, effectively minimizing the potential impact of local horizontal pleiotropy that often masks or biases causal effects in typical univariable MR estimates. Consequently, this approach provides more rigorous statistical insight into whether the causal protective effect on AD via LDL-c reduction remains robustly significant when adjusted for concurrent HbA1c levels and, conversely, whether the causal effect via HbA1c reduction remains significant when adjusting for LDL-c levels. Similarly, extending this identical cis-MVMR methodology to evaluate DR and CAD yields critical insights into the distinct molecular basis of these vascular complications. For DR, this multivariable approach clarifies the relative causal contributions of glycemic versus lipidomic pathways in preserving retinal microvascular health. For CAD, it delineates the independent macrovascular impacts of specific metabolic mediators, isolating the atheroprotective effects of lipid modulation from those of glycemic control or weight reduction. Ultimately, deploying this unified analytical framework across AD, DR, and CAD allows us to comprehensively map how the highly correlated, pleiotropic effects of dual incretin agonism translate into distinct, tissue-specific neurovascular, microvascular, and macrovascular benefits in treated T2D patients.

### 2.2. Data Sources

All datasets utilized are publicly accessible and summarized in Table 1.

As displayed in Table 1, summary-level genetic association data for relevant biomarkers and anthropometric traits, including glycated hemoglobin (HbA1c), low-density lipoprotein cholesterol (LDL-c), high-density lipoprotein cholesterol (HDL-c), and body mass index (BMI), were obtained from the Pan UK Biobank European ancestry cohort [41]. Summary statistics for disease outcomes were derived from large-scale, publicly available genome-wide association studies (GWASs). Data for Alzheimer’s disease (AD) and AD-by-proxy (family history) were sourced from the meta-analysis by Schwartzentruber et al. (N=472,868; 75,024 cases) [42]. Diabetic retinopathy (DR) data were obtained from Verma et al. (N=432,209; 29,668 cases) [43], type 2 diabetes (T2D) from Xue et al. (N=659,316; 62,892 cases) [44], and coronary artery disease (CAD) from Aragam et al. (N=1,165,690; 181,522 cases) [45]. Linkage disequilibrium (LD) patterns were estimated using a reference panel of 882 European individuals from the 1000 Genomes Project Phase III [46].

### 2.3. Instrumental Variable (IV) Selection

We selected genetic instruments defined as single-nucleotide polymorphisms (SNPs) located within ±100 kb of the *GLP1-R* and *GIPR* gene loci. For harmonization, SNPs were required to be present across the exposure(s), outcome and LD reference datasets. We excluded variants with an effect allele frequency (EAF) < 1% or a difference in EAF > 5% across GWAS datasets for quality control purposes and applied Steiger filtering to prevent bias from reverse causality. Genetic proxies were selected based on a significance threshold of p<0.01 in their associations with the exposure trait (or either of the exposure traits if applicable), and an LD pruning threshold of $r^{2}<0.9$ was applied to ensure the adequate coverage of genetic variation within the target regions while avoiding excessive collinearity. To evaluate the potential synergistic effects of dual incretin receptor targeting, we constructed a combined dual agonism model by pooling the selected instrumental variables from both the *GLP1R* and *GIPR* loci into a single instrument variable set. This combined dual agonism model allows us to genetically proxy the concurrent pharmacological activation of both incretin receptors, providing a framework to assess their complementary therapeutic impact in a manner that mirrors the mechanism of emerging dual agonist therapies.

### 2.4. The cis-MR PC-GMM Method

Conventional Mendelian randomization (MR) utilizes genome-wide instrumental variable selection, which increases vulnerability to horizontal pleiotropy related biases. To mimic targeted pharmacological perturbation and minimize pleiotropic biases, we adopted a cis-MR approach [47,48] by restricting the selection of instrumental variables (IVs) strictly to the target loci (*GLP1R* and *GIPR*) [33]. We first applied univariable cis-MR (cis-UVMR) to estimate the marginal effects of discrete metabolic mediators. To disentangle the effects of correlated mediators (HbA1c and LDL-c), we extended our framework to multivariable cis-MR (cis-MVMR), which concurrently models multiple potential pathways to account for local horizontal pleiotropy. Because variants within a single cis-region exhibit high linkage disequilibrium (LD), standard LD clumping discards substantial genetic variance. This severe reduction in instruments not only exacerbates weak instrument bias but can diminish power of MR and render MR models mathematically unidentifiable (e.g., leaving fewer independent instruments than exposures). Conversely, retaining these highly correlated variants in standard models induces severe multicollinearity and numerical instability [49].

To resolve these challenges, we employed the Principal Component Generalized Method of Moments (PC-GMM) estimator [33]. The PC-GMM method projects *M* correlated genetic variants onto an orthogonal matrix Λ, which comprises the first *L* principal components of the weighted genetic correlation matrix (capturing ≥99% variance). This transformation yields dimension-reduced, orthogonal summary associations for the exposures ($\hat{\gamma }$) and the outcome ($\hat{\Gamma }$), effectively avoiding multicollinearity. The MR effect vector θ (of length *K* for *K* exposures) is then estimated by minimizing the continuously updating GMM criterion function $\hat{Q}\left(\theta ,\kappa^{2}\right)$ [50,51]:

$$\hat{Q}\left(\theta ,\kappa^{2}\right)=\hat{g}{\left(\theta \right)}^{\prime }\hat{\Omega }{\left(\theta ,\kappa^{2}\right)}^{-1}\hat{g}\left(\theta \right),$$

where the empirical moment conditions are defined as $\hat{g}\left(\theta \right)=\hat{\Gamma }-\hat{\gamma }\theta$, and $\hat{\Omega }\left(\theta ,\kappa^{2}\right)$ is the optimal weighting matrix [33]. Crucially, this framework incorporates an overdispersion variance parameter, $\kappa^{2}$, directly into the weighting matrix to account for the extra uncertainty introduced by random direct (pleiotropic) effects [33]. By iteratively updating $\hat{\Omega }$ and rescaling the moment variances with this parameter [50,51], the model mitigates weak instrument bias while preventing the estimates from being misleadingly over-precise.

## 3. Results

### 3.1. Positive Control Analyses Using cis-MR: Synergistic Causal Effect of Dual Agonism for T2D

To validate the selected IVs and the robust PC-GMM-based cis-MR analysis, we performed positive control analyses leveraging the established clinical relationship between HbA1c and T2D to assess the synergistic significant causal association between GLP-1R/GIPR agonism and risk reduction in T2D via HbA1c control. Specifically, we used a two-sample univariable cis-Mendelian randomization (cis-UVMR) analysis to examine this expected causality, using genetic variations within the *GLP1-R* and *GIPR* regions as drug target proxies. The IV selection process identified 80 and 11 SNPs within the *GLP1-R* and *GIPR* loci, respectively, yielding 91 SNPs for the combined dual agonism model, which were significantly associated with alterations in HbA1c levels. Using principal components accounting for 99% of the weighted genetic variation, we found that a genetically predicted one-standard-deviation decrease in HbA1c at the *GLP1-R* locus corresponded to a significant decrease in T2D risk (OR = 0.26; 95% CI, 0.15–0.45; $p=2.14\times 10^{-6}$), as displayed in Table 2. A similar and even more pronounced effect was observed at the *GIPR* locus (OR = 0.14; 95% CI, 0.08–0.26; $p=6.36\times 10^{-11}$). Crucially, evaluating the combined *GLP1R*/*GIPR* dual agonism model yielded a highly robust protective association (OR = 0.17; 95% CI, 0.11 0.26; $p=3.68\times 10^{-17}$) which is more significant than either individual agonism alone in support of the observed clinical synergy [15].

These novel findings, summarized in Table 2, demonstrate that both incretin-related genetic proxies effectively capture the expected diagnostic and mechanistic pathways of T2D management. Furthermore, they support the hypothesis of the synergistic causal effect of dual incretin targeting from a genetically proxied perspective, confirming that HbA1c control remains a central mechanism through which these pathways modulate the risk of T2D as a common metabolic disease.

### 3.2. Univariable cis-Mendelian Randomization for AD, DR and CAD

We implemented a univariable cis-Mendelian Randomization (cis-UVMR) approach using the robust PC-GMM estimator to identify potential causal pathways linking the *GLP1-R* and *GIPR* loci to Alzheimer’s disease (AD), diabetic retinopathy (DR) and coronary artery disease (CAD). For AD and DR, HbA1c and LDL-c were used as mediating biomarkers of dual agonism as these represent the primary glycemic and lipidomic pathways through which incretin mimetics are clinically expected to exert neurovascular protection.

**Figure 2:**
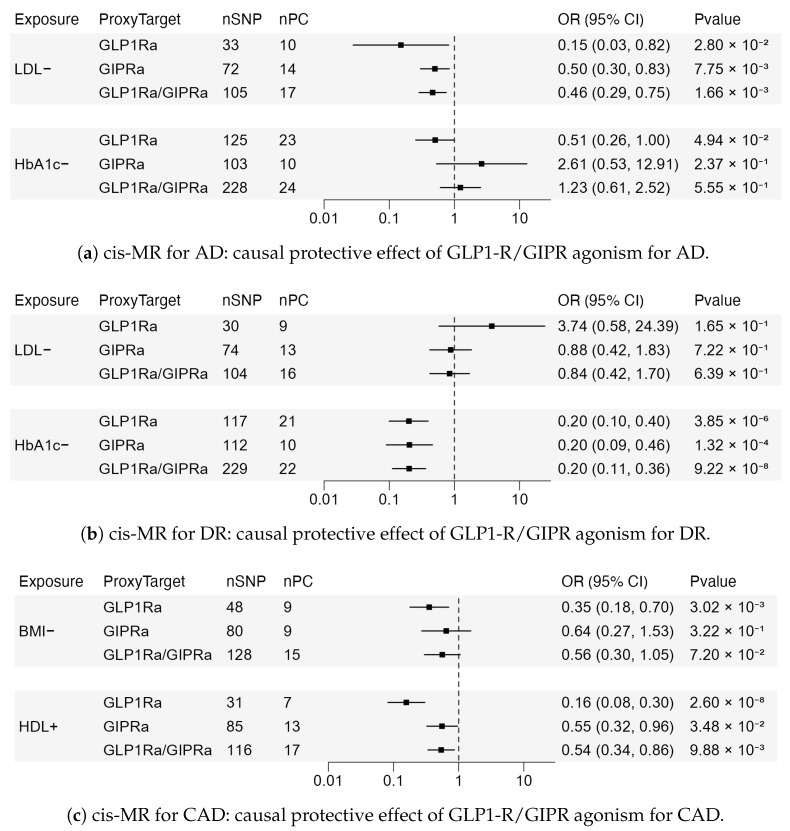
Univariable Mendelian randomization (cis-UVMR) analysis of genetically predicted dual-targeting drug effect on disease risk via pathways involving multiple biomarkers. Estimates were derived via robust PC-GMM estimation using principal components accounting for 99% of weighted genetic variation at *GLP1-R*/*GIPR* loci. (**a**) cis-UVMR estimates of genetically proxied drug effect on Alzheimer’s disease (AD) via reduced LDL or HbA1c level. (**b**) cis-UVMR estimates of genetically proxied drug effect on diabetic retinopathy (DR) via reduced LDL or HbA1c level. (**c**) cis-UVMR estimates of genetically proxied drug effect on coronary artery disease (CAD) via reduced BMI or elevated HDL level. nSNP: number of single-nucleotide polymorphisms utilized as instrumental variables; nPC: number of principal components retaining 99% of genetic variance.

As illustrated in Figure 2, the univariable cis-MR estimates revealed a distinct causal mechanism for AD and DR. For Alzheimer’s disease (Figure 2a), genetically predicted LDL-c reduction at both the *GLP1-R* locus (OR = 0.15; 95% CI, 0.03–0.82; p=0.028) and the *GIPR* locus (OR = 0.50; 95% CI, 0.30–0.83; p=0.008), as well as in the combined *GLP1R*/*GIPR* dual agonism model (OR = 0.46; 95% CI, 0.29–0.75; $p=1.66\times 10^{-3}$), demonstrated significant protective causal associations. Conversely, the glycemic pathway via HbA1c showed a marginal association at the *GLP1-R* locus (p=0.049) and was non-significant at the *GIPR* locus (p=0.237) and in the combined dual agonism model (p=0.555). In contrast, for diabetic retinopathy (Figure 2b), the causal signals were predominantly driven by the glycemic pathway. Genetically predicted HbA1c levels were strongly associated with DR risk at both the *GLP1-R* locus (OR = 0.20; 95% CI, 0.10–0.39; $p=3.85\times 10^{-6}$) and the *GIPR* locus (OR = 0.20; 95% CI, 0.09–0.46; $p=1.32\times 10^{-4}$), as well as in the combined *GLP1R*/*GIPR* dual agonism model (OR = 0.20; 95% CI, 0.11–0.36; $p=9.22\times 10^{-8}$). In contrast to AD, the association of LDL-c did not reach statistical significance for DR at either locus (p=0.165 and p=0.722, respectively) nor in the combined dual agonism model (p=0.639). Additionally, to investigate the potential shared pathological mechanism, we applied cis-UVMR to model genetically proxied DR risk reduction as the exposure and AD as the outcome. At the *GLP1-R* locus, a genetically proxied reduction in DR risk demonstrated a statistically significant causal association with a decreased risk of AD (OR = 0.77; 95% CI, 0.60–0.99; p=0.047). This is consistent with the finding that DR predicts the risk of AD from the Danish registry-based nationwide cohort study [2].

For coronary artery disease (Figure 2c), protective causal signals were predominantly driven by the HDL pathway. Genetically proxied HDL levels demonstrated a robust and significant protective association with CAD across all investigated targets: the *GLP1-R* locus (OR = 0.16; 95% CI, 0.08–0.30; *p* = 2.60 × 10^−8^), the *GIPR* locus (OR = 0.55; 95% CI, 0.32–0.96; *p* = 0.035), and the combined GLP1-R/GIPR dual agonism model (OR = 0.54; 95% CI, 0.34–0.86; *p* = 0.009). Conversely, when examining the impact of the anthropometric pathway via BMI, a significant protective causal effect was strictly isolated to the *GLP1-R* locus (OR = 0.35; 95% CI, 0.18–0.70; *p* = 0.003). The BMI-mediated effect was non-significant at the *GIPR* locus (OR = 0.64; 95% CI, 0.27–1.53; *p* = 0.322) and narrowly missed statistical significance in the combined dual agonism model (OR = 0.56; 95% CI, 0.30–1.05; *p* = 0.072).

### 3.3. Multivariable cis-Mendelian Randomization for AD, DR and CAD

To disentangle the respective causal contributions of glycemic and lipidomic pathways, and to identify whether specific pathways remain significant when adjusting for the other, we conducted a multivariable cis-Mendelian randomization (cis-MVMR) analysis. While the preceding UVMR provided a view of individual biomarker effects, the MVMR framework is statistically superior in this context as it accounts for the inherent genetic correlation between HbA1c and LDL (or between HDL and BMI) within the *GLP1-R* and *GIPR* loci. By evaluating these exposures simultaneously, MVMR identifies the “direct” effect of each mediator, effectively adjusting for horizontal pleiotropy that often masks or biases univariable estimates from conventional univariate MR analysis. The genetic association for the instrumental variables used in the MVMR analyses are summarized in Supplementary Material.

In the MVMR model for AD (Table 3a), the significance of the lipidomic pathway was further refined and strengthened. At the *GLP1-R* locus, once adjusted for HbA1c, the decrease in LDL remained a significant driver of reduced AD risk (OR = 0.13; 95% CI = (0.03, 0.70); p=0.017). Notably, the adjusted effect of HbA1c reduction was also significant (OR = 0.47; 95% CI = (0.23, 0.95); p=0.036), revealing that the protective effect of GLP1-R agonism on AD is a dual-mechanism process involving both metabolic stabilization and lipid management. At the *GIPR* locus, the lipidomic signal remained significant (OR = 0.41; 95% CI = (0.18, 0.93); p=0.033), whereas the HbA1c effect was non-significant (p=0.858), suggesting that the dominant protective effect of GIPR agonism alone on AD is through the LDL-involved pathway. Similarly, in the combined *GLP1R*/*GIPR* dual agonism model, the protective effect against AD was exclusively driven by the lipidomic pathway (OR = 0.44; 95% CI = (0.25, 0.81); p=0.007), while the adjusted effect of HbA1c was non-significant (p=0.659). Not much evidence of synergy was observed.

In the MVMR model for DR (Table 3b), at the *GLP1-R* locus, the primary driver for DR risk reduction remained HbA1c (OR = 0.16; 95% CI = (0.07, 0.36); $p=8.63\times 10^{-6}$), confirming that glycemic control is a very potent mechanism for preserving retinal microvascular integrity, while the LDL pathway did not reach statistical significance (p=0.217) (Table 3b). At the *GIPR* locus, the glycemic pathway through HbA1c showed a strong causal association with DR (OR = 0.18; 95% CI = (0.06, 0.53); $p=1.62\times 10^{-3}$), while the LDL pathway did not reach statistical significance (p=0.098). This suggests that with either GLP1R agonism alone or GIPR agonism alone, protection against microvascular complications in the retina, DR, is primarily governed by glycemic stability rather than lipid modulation. The LDL effect is negligible in each agonism alone when adjusted for HbA1c. Interestingly, when evaluating the combined *GLP1R*/*GIPR* dual agonism model, both pathways emerged as significant drivers of reduced DR risk. The glycemic pathway maintained a highly significant protective association (OR = 0.18; 95% CI = (0.10, 0.34); $p=4.10\times 10^{-8}$), and the lipidomic pathway also achieved statistical significance (OR = 0.53; 95% CI = (0.29, 0.94); p=0.031). This is consistent with the notion that the synergistic effect of lipid modulation is dual agonism.

In the multivariable model for CAD (Table 3c), at the *GLP1-R* locus, neither BMI (p=0.808) nor HDL (p=0.069) retained a statistically significant causal association after adjusting for the alternative pathway. This means that the joint protective effect of GLP-1R agonism alone is not significant. Similarly, at the *GIPR* locus, the effect of HDL was non-significant (p=0.078) for CAD when adjusted for genetically proxied BMI reduction, which emerged as a significant protective effect of reduced CAD risk (OR = 0.48; 95% CI = (0.25, 0.92); p=0.028). Remarkably, in the combined *GLP1-R*/*GIPR* dual agonism model, both pathways demonstrated robust protective associations. Genetically proxied BMI reductions (OR = 0.46; 95% CI = (0.28, 0.75); $p=2.27\times 10^{-3}$) and HDL improvements (OR = 0.59; 95% CI = (0.39, 0.90); p=0.013) both significantly lowered CAD risk. This indicates the existence of synergistic macrovascular protection afforded by dual incretin agonism through compounded respective improvements in both weight management and lipid metabolism.

## 4. Discussion

Dual-targeting antidiabetic drugs glucagon-like peptide-1 receptor (GLP-1R) and glucose-dependent insulinotropic polypeptide receptor (GIPR) agonists have demonstrated remarkable clinical efficacy in lowering glycated hemoglobin (HbA1c) and controlling body weight and BMI [15,16]. In addition, these agents also improve broader metabolic profiles, notably by reducing low-density lipoprotein cholesterol (LDL-c) and increasing high-density lipoprotein cholesterol (HDL-c) [35,36,37,38,39,40]. These metabolic improvements overlap with the top known modifiable factors for dementia [26] and protective factors for cardiovascular diseases (CVD) [52]. Given that dementia and CVD are key drivers of the main morbidity and mortality among T2D patients, there is great public health significance in investigating whether the neuroprotective, micro- and macrovascular benefits of these dual agonists are significantly causally mediated through shared metabolic modulation [27,53].

To address the currently unmet needs, we used a cis-UVMR and cis-MVMR approach based on robust PC-GMM to establish that the causal protective effects of the dual agonism of GLP1R/GIPR on T2D, DR, CAD, and AD indeed all act in the same protective direction as a necessary molecular basis (though not a sufficient one) for showing that GLP1R agonism and GIPR agonism work together synergistically: (1) The positive control cis-MR analyses confirmed a synergistic causal protective effect of dual GLP-1R/GIPR agonism on T2D via HbA1c reduction [OR = 0.1; 95% CI = (0.11, 0.26); *p* = $3.68\times 10^{-17}$], which is more significant than that of either GLP-1R agonism or GIPR agonism alone. (2) The multivariate cis-MR (or cis-MVMR) analyses revealed that after adjusting for HbA1c, a synergistic protective effect on DR via a reduction in LDL-c is significant in dual GLP-1R/GIPR agonism [OR = 0.57; 95% CI = (0.29, 0.94)], while the protective effect on DR of LDL-c reduction is non-significant in either GLP-1R agonism or GIPR agonism alone. (3) After adjusting for HbA1c, the multivariate cis-MR results showed significant protective effects on AD via a reduction in LDL-c in GLP-1R/GIPR agonism [OR = 0.44; 95% CI = (0.25, 0.81)]. The causal protective effect of dual GLP-1R/GIPR agonism via HbA1c reduction was also significant for DR [OR = 0.20; 95% CI = (0.11, 0.36); *p* = $9.22\times 10^{-8}$]. In turn, the cis-UVMR analysis of GLP1R agonism also showed that a genetically proxied reduction in DR risk demonstrated a significant causal association with a decreased risk of AD [OR = 0.77; 95% CI = (0.60, 0.99); *p* = 0.047]. (4) Importantly, the multivariate cis-MR results also revealed that dual GLP-1R/GIPR agonism has significant protective effects on CAD via both a reduction in BMI [OR = 0.46; 95% CI = (0.28, 0.75)] and an improvement in HDL [OR = 0.59; 95% CI = (0.39, 0.90)]. This is in support of the hypothesis that dual GLP-1R/GIPR agonism has a synergistic protective effect on CAD that is stronger than that of GLP-1R agonism alone, which yielded a non-significant causal effect for both HDL and BMI, and also stronger than that of GIPR agonism alone, which yielded a non-significant causal effect for HDL when adjusted for BMI.

These novel findings have significant implications for repurposing dual incretin agonism in terms of diabetic drugs to serve as a unifying, precision prevention strategy against CAD, DR and AD as leading drivers of mortality and morbidity in diabetic patients.

These novel results on synergistic causal effects on DR, AD and CAD via dual GLP-1R/GIPR agonism in patients receiving antidiabetic therapy are strongly supported by a growing body of mechanistic and animal-based literature detailing the direct neuroprotective effects of incretin-based therapies. Both GLP-1 and GIP receptors are expressed in key brain regions relevant to cognition, including the hippocampus and cortex [21,54,55]. Their agonists are capable of penetrating the blood–brain barrier, albeit with varying efficiency, to exert central effects that can occur independently of systemic glycemic or lipidic control [22,56,57]. Preclinical models of Alzheimer’s disease demonstrate that GLP-1R agonism reduces neuroinflammation by modulating microglial activation and inhibiting the formation of reactive astrocytes, thereby decreasing oxidative stress and neuronal apoptosis [58,59]. Incretin signaling also counters central insulin resistance by improving cerebral glucose metabolism and mitochondrial function [21,22,60]. Furthermore, experimental studies show that GIPR agonism supports neurogenesis and synaptic plasticity, while dual GLP-1/GIP receptor activation produces complementary benefits, including reductions in amyloid-β (Aβ) plaque burden and tau hyperphosphorylation [21,24,61,62]. Therefore, our genetic evidence that dual incretin receptor perturbation mitigates AD risk aligns robustly with these established biological mechanisms. This suggests that the systemic benefits we modeled (HbA1c and LDL-c reduction) are likely augmented by direct, concurrent neurotrophic signaling within the central nervous system.

The discrepancy in the causal estimates for HbA1c reduction on Alzheimer’s disease (AD) risk between the GLP1R locus and the GIPR locus likely reflects the distinct biological roles of these two receptors. While the agonism of both targets effectively lowers systemic HbA1c, their downstream signaling cascades within the central nervous system differ. The data suggest a therapeutic “division of labor”: while both contribute fundamentally to metabolic health, GLP-1Ra serves as the primary engine for glycemic and lipidomic neuroprotection to reduce the risk of AD, whereas GIPRa provides an LDL-mediated synergy. This is boosted by the potent protective effect of both GLP1-Ra and GIPRa on DR via HbA1c. The shared causal mediation of AD risk through LDL-c reduction is a novel finding and particularly noteworthy, providing genetic corroboration for the 2024 Lancet Commission’s emphasis on lipid management as a top-tier modifiable risk factor for dementia [26]. It is known that AD is the most common cause of dementia [8]. By reducing systemic LDL-c, these therapies significantly reduce the risk of cardiovascular disease (CVD, a leading cause of mortality) and mitigate vascular-driven neurodegeneration, which is the most common subtype of neuropathology in AD [8]. Also, our cis-MR analyses (Table 3c) indicated that dual GLP-1R and GIPR agonism causally reduces the risk of coronary artery disease (CAD), the most common type of CVD, via distinct processes involving boosting HDL-c [OR = 0.59; 95% CI = (0.39, 0.90); p=0.013] and controlling BMI [OR = 0.46; 95% CI = (0.28, 0.75); *p* = 0.0023]. This is of clinical significance given that heart disease has been the leading cause of death in the US and globally.

Concurrently, robust glycemic protection against DR can be contextualized through the restoration of proteostasis. As reviewed by Huang et al. [63], chronic hyperglycemia exacerbates endoplasmic reticulum (ER) stress, disrupts retinal redox balance and calcium signaling, and leads to maladaptive unfolded protein response (UPR) activation. Incretin signaling appears to counter this by fine-tuning the PERK and IRE1α pathways, alleviating retinal dysfunction, thus presenting an effective way to prevent AD in persons with diabetes.

The primary strength of this study lies in the application of a novel, robust principal component-based generalized method of moments (PC-GMM) estimator within a multivariable cis-Mendelian randomization framework recently developed by Patel et al. [33]. This cis-MVMR approach restricts instruments to drug target coding regions, effectively limiting pleiotropic effects while mimicking pharmacological perturbation, and enables the simultaneous evaluation of dissected causal effects for correlated metabolic mediators like HbA1c and LDL-c. Additionally, the inclusion of DR as a secondary outcome provides unique biological validation; the consistent protective signals across both the retina and the brain substantiate the neuroprotective potential of dual incretin targeting and reinforce the role of the retina as a non-invasive window into cerebral microvascular integrity. Importantly, multivariable analyses from a large, population-based Danish national cohort [2] demonstrated that those with diabetes but no DR had a reduced risk of developing AD [adjusted HR 0.87, 95% CI = (0.81, 0.93)] compared to individuals without diabetes. Conversely, individuals with both diabetes and DR exhibited a higher risk of AD [adjusted HR 1.24, 95% CI = (1.08, 1.43)]. When individuals with diabetes and no DR were utilized as the reference group, a significantly higher risk of incident AD was observed in those with DR [adjusted HR 1.34, 95% CI = (1.18, 1.53)]. The robustness of these observational findings is underscored by the study’s substantial sample size and its prospective, longitudinal design. These findings suggest that preventing DR is an effective way to prevent AD. Therefore, our findings from robust cis-MR analysis on DR and AD carry significant clinical implications for the precision prevention of AD, which currently has no cure.

Before further discussion on the methodology and other methodological strengths, several limitations warrant consideration. First, all data used in our study are from publicly available databases (as listed in Table 1); therefore, data quality and statistical power may be constrained by the inclusion criteria and sample sizes of the original studies. In particular, this may have limited the power of the cis-MR reported in Figure 2c. Second, the datasets relied on blood-based GWASs rather than retinal, brain or heart tissue GWASs. Gene expression differs by tissue, which could affect the selection and validity of the instrumental variables used for the cis-MR analyses. Third, our analysis was restricted to individuals of European ancestry to minimize population stratification, potentially limiting the generalizability of the findings to more diverse global populations. Finally, the possibility of false-positive findings in our genetic causal inference must be carefully considered. Although our cis-MVMR framework explicitly adjusts for measured pleiotropy to satisfy the critical exclusion restriction assumption, the potential for unmeasured horizontal pleiotropy via unknown, independent biological pathways cannot be entirely ruled out. If these core instrumental variable assumptions are violated, the validity of the MR estimates is undermined, directly increasing the risk of false positives. Furthermore, because our study evaluates multiple metabolic exposures across distinct genetic loci against multiple disease outcomes, there is an inherent risk of false-positive findings driven by multiple comparisons.

Despite the above-mentioned limitations, this research exemplifies how the robust cis-MVMR design serves as a highly promising screening instrument in the drug development phase, allowing for the rigorous testing of interventions and the discovery of new indications for approved drugs in a manner that bypasses some time and cost constraints of traditional trials. It provides robust supporting evidence that genetic variation within the antidiabetic drug target genes *GLP1-R* and *GIPR* are causally associated with a lower risk of DR and AD through HbA1c and LDL-c control, in addition to a lower risk of CAD through HDL-c and BMI improvement. Given that up to 20–30% of diabetic patients have DR globally [64], the synergistic clinical efficacy of GIPR/GLP-1R agonists against DR is likely a dominant contributor to the overarching reduction in AD risk [2,65], positioning these dual-agonist therapies as a holistic, precision medicine strategy to mitigate the global burden of both vision loss and cognitive impairment. Additionally, given that LDL-c/HDL-c are the most prominent risk factors for CVD, which has been the leading cause of death in the USA and many other populations, the significant reduction in LDL-c and enhancement in HDL-c by dual GLP-1R/GIPR agonism in T2D can potentially lead to a significant reduction in CVD-related mortality.

Finally, our findings demonstrate that the favorable effects of dual GLP1R and GIPR agonism on lipid profile improvement, glycemic regulation, and weight control position this class of drugs to simultaneously address multiple major complications of type 2 diabetes. Rather than acting through a singular pathway, the complementary mechanisms of HbA1c and LDL-c reduction highlight an integral, precision-based therapeutic profile capable of mitigating the risks of Alzheimer’s disease and diabetic retinopathy, alongside concurrent BMI and HDL-c improvements that protect against cardiovascular disease. Ultimately, these data underscore the profound potential for dual incretin agonism to serve as a unifying, precision prevention strategy against the leading drivers of morbidity and mortality in high-risk diabetic populations.

## Figures and Tables

**Table 1:** Data sources used in this study.

GWAS Data Source	Phenotype	Sample Size	N_Case *
Pan-UK Biobank—EUR [41]	HbA1c (field 30750)	400,825	-
	LDL (field 30780)	400,223	-
	HDL (field 30760)	367,021	-
	BMI (field 21001)	419,163	-
Schwartzentruber et al. [42]	AD/family history	472,868	75,024
Verma et al. [43]	DR	432,209	29,668
Xue et al. [44]	T2D	659,316	62,892
Aragam et al. [45]	CAD	1,165,690	181,522
1000 Genomes—Phase III EUR [46]	LD reference panel	882	-

* Abbreviations: HbA1c: glycated hemoglobin; LDL: low-density lipoprotein; HDL: high-density lipoprotein; BMI: body mass index; AD: Alzheimer’s disease; DR: diabetic retinopathy; T2D: type 2 diabetes; CAD: coronary artery disease; LD: linkage disequilibrium; N_case: number of cases.

**Table 2:** **cis-MR for T2D.** Univariable cis-Mendelian randomization (cis-UVMR) analysis of genetically predicted dual-targeting drug effect on T2D via HbA1c reduction. Estimates in cis-MR were derived via robust PC-GMM estimation using principal components accounting for 99% of weighted genetic variation at *GLP1-R*, *GIPR* and combined *GLP1-R*/*GIPR* loci respectively.

ProxyTarget	Exposure	Estimate (SE) *	OR (95% CI)	*p*-Value	nSNP	nPC
GLP1Ra	HbA1c−	−1.35 (0.28)	0.260 (0.149, 0.454)	$2.14\times 10^{-6}$	80	17
GIPRa	HbA1c−	−1.95 (0.30)	0.142 (0.079, 0.256)	$6.36\times 10^{-11}$	11	5
GLP1Ra/GIPRa	HbA1c−	−1.78 (0.21)	0.169 (0.112, 0.256)	$3.68\times 10^{-17}$	91	21

* Abbreviations: Estimate (SE): cis-MR effect estimate with standard error; OR = exp (Estimate); OR: odds ratio per one standard deviation reduction in genetically proxied HbA1c levels at the *GLP1R*/*GIPR* loci; nSNP: number of single-nucleotide polymorphisms utilized as instrumental variables; nPC: number of principal components retaining 99% of the genetic variance.

**Table 3:** Multivariable Mendelian randomization (cis-MVMR) analysis of genetically predicted dual-targeting drug effect on disease onset via LDL/HbA1c-mediated pathways. Estimates were derived via robust PC-GMM estimation using principal components accounting for 99% of weighted genetic variation at *GLP1-R*/*GIPR* loci. (**a**) Cis-MVMR estimates of genetically proxied drug effect on Alzheimer’s disease via LDL (LDL−) and HbA1c reduction (HbA1c−). (**b**) Cis-MVMR estimates of genetically proxied drug effect on diabetic retinopathy via LDL (LDL−) and HbA1c reduction (HbA1c−). (**c**) Cis-MVMR estimates of genetically proxied drug effect on coronary artery disease via BMI reduction (BMI−) and HDL elevation (HDL+).

(a)cis-MVMRforAD							
**ProxyTarget**	**Exposure**	**Estimate**	**SE**	OR * (95% CI)	**p-Value**	**nSNP**	**nPC**
GLP1Ra	LDL−	−2.01	0.84	0.13 (0.03, 0.70)	0.017	151	24
	HbA1c−	−0.76	0.36	0.47 (0.23, 0.95)	0.036		
GIPRa	LDL−	−0.89	0.42	0.41 (0.18, 0.93)	0.033	135	16
	HbA1c−	−0.11	0.61	0.90 (0.27, 2.95)	0.86		
GLP1Ra/GIPRa	LDL−	−0.81	0.30	0.44 (0.25, 0.81)	0.007	286	30
	HbA1c−	−0.14	0.32	0.87 (0.46, 1.64)	0.66		
**(b) cis-MVMR for DR**							
**ProxyTarget**	**Exposure**	**Estimate**	**SE**	**OR (95% CI)**	**p-Value**	**nSNP**	**nPC**
GLP1Ra	LDL−	−1.20	0.97	0.30 (0.05, 2.02)	0.217	140	21
	HbA1c−	−1.81	0.41	0.16 (0.07, 0.36)	$8.63\times 10^{-6}$		
GIPRa	LDL−	−0.62	0.37	0.54 (0.26, 1.12)	0.098	148	15
	HbA1c−	−1.71	0.54	0.18 (0.06, 0.53)	$1.62\times 10^{-3}$		
GLP1Ra/GIPRa	LDL−	−0.64	0.30	0.53 (0.29, 0.94)	0.031	288	28
	HbA1c−	−1.70	0.31	0.18 (0.10, 0.34)	$4.10\times 10^{-8}$		
**(c) cis-MVMR for CAD**							
**ProxyTarget**	**Exposure**	**Estimate**	**SE**	**OR (95% CI)**	**p-Value**	**nSNP**	**nPC**
GLP1Ra	BMI−	0.23	0.95	1.26 (0.20, 8.03)	$8.08\times 10^{-1}$	74	12
	HDL+	−1.45	0.80	0.23 (0.05, 1.12)	$6.86\times 10^{-2}$		
GIPRa	BMI−	−0.74	0.34	0.48 (0.25, 0.92)	$2.76\times 10^{-2}$	122	12
	HDL+	−0.50	0.28	0.61 (0.35, 1.06)	$7.80\times 10^{-2}$		
GLP1Ra/GIPRa	BMI−	−0.79	0.26	0.46 (0.28, 0.75)	$2.27\times 10^{-3}$	196	19
	HDL+	−0.52	0.21	0.59 (0.39, 0.90)	$1.32\times 10^{-2}$		

* Abbreviations: Estimate: cis-Mendelian randomization effect estimate for the corresponding exposure, adjusted for the alternative exposure trait; OR: odds ratio per one-standard-deviation reduction in genetically proxied LDL or HbA1c levels at the *GLP1R*/*GIPR* loci, adjusted for the alternative exposure trait; nSNP: number of single-nucleotide polymorphisms utilized as instrumental variables; nPC: number of principal components retaining 99% of the genetic variance.

## Data Availability

The original data presented in the study are openly available in the public repositories and GWAS consortia listed in Table 1. This includes metabolic and anthropometric phenotypes from the Pan-UK Biobank [41], as well as summary statistics for Alzheimer’s disease (Schwartzentruber et al. [42]), type 2 diabetes (Xue et al. [44]), and diabetic retinopathy (Verma et al. [43]). All linkage disequilibrium calculations were performed using the 1000 Genomes Project Phase III European reference panel [46].

## Supplementary Materials

The following supporting information can be downloaded at: https://www.mdpi.com/article/10.3390/genes17060602/s1.

## Abbreviations

The following abbreviations are used in this manuscript:

A*β*	Amyloid Beta
AD	Alzheimer’s Disease
BMI	Body Mass Index
cis-MR	Cis-Mendelian Randomization
CAD	Coronary Artery Disease
CVD	Cardiovascular Disease
DR	Diabetic Retinopathy
ER	Endoplasmic Reticulum
GIPR	Glucose-Dependent Insulinotropic Polypeptide Receptor
GIPRa	Glucose-Dependent Insulinotropic Polypeptide Receptor Agonism
GLP-1R	Glucagon-Like Peptide-1 Receptor
GLP-1Ra	Glucagon-Like Peptide-1 Receptor Agonism
GMM	Generalized Method of Moments
GWAS	Genome-Wide Association Study
HbA1c	Glycated Hemoglobin
HDL/HDL-c	High-Density Lipoprotein Cholesterol
IPTW	Inverse Probability of Treatment Weighting
IRE1α	Inositol-Requiring Enzyme 1 Alpha
IV	Instrumental Variable
LDL/LDL-c	Low-Density Lipoprotein Cholesterol
MR	Mendelian Randomization
MVMR	Multivariable Mendelian Randomization
PC	Principal Component
PERK	Protein Kinase RNA-Like Endoplasmic Reticulum Kinase
SNV	Single-Nucleotide Variant
T2D/T2DM	Type 2 Diabetes Mellitus
UPR	Unfolded Protein Response
UVMR	Univariable Mendelian Randomization
